# Incidentally Diagnosed COVID-19 Infection in Trauma Patients; a Clinical Experience

**Published:** 2020-03-23

**Authors:** Mehdi Khazaei, Reyhaneh Asgari, Ehsan Zarei, Yashar Moharramzad, Hamidreza Haghighatkhah, Morteza Sanei Taheri

**Affiliations:** 1 Department of Radiology, Shahid Beheshti University of Medical Sciences, Tehran, Iran

**Keywords:** Coronavirus, COVID-19, Tomography, X-Ray Computed, wounds and injuries, safety

**Dear Editor,**

The novel coronavirus disease (COVID-19) has rapidly spread across the world and caused a pandemic, and still continues to evolve. In Iran, the first cases of COVID-19 were officially announced between February 19 and 23, 2020 and it soon became clear that Iran is one of the countries that is worst-hit by COVID-19 outbreak (1, 2).

It is now evident that most cases of COVID-19 disease develop mild respiratory and constitutional symptoms (3), while some cases are asymptomatic (3, 4). Involvement of other organs, including liver and kidneys has been reported in patients with COVID-19 (5). Many questions remain unanswered about associations and presentations of COVID-19. 

Shohada-e-Tajrish Hospital, a university hospital located in Tehran, Iran, was designated as one of the main centers providing diagnostic and healthcare services for patients suspected to be infected with the new virus. Here, we share our experience regarding computed tomography (CT) findings suggestive of COVID-19 disease among patients who underwent radiologic investigation due to traumatic injuries. 

Several trauma patients had findings on their chest and/or abdominal CT scans, which were suggestive or highly suggestive of COVID-19 (1). The findings were noted in the lung bases that were visible on an abdominal CT scan or on a clinically indicated spiral CT scan of the chest. The most frequent mechanisms of injury were fall and car accident. Conscious patients did not report dizziness or loss of consciousness before the trauma and most of them did not have any symptoms related to COVID-19 (e.g., fever, shortness of breath). To our knowledge, there is no study or report about the possible association between COVID-19 and trauma in the literature. So, the mentioned observations could be just a coincidence, but this association could still be proposed as a research objective. The importance of this finding is that CT characteristics of COVID-19 could be seen in patients who were admitted to the hospitals not due to COVID-19 related symptoms, but because of chest and abdominal trauma. This issue should be considered by health care workers serving trauma patients and it should be kept in mind that during an outbreak of respiratory infections, in particular COVID-19 disease, every attempt should be made to protect the health and safety of the staff even when providing care for trauma patients.
